# Nomogram for evaluating obvious liver inflammation in treatment-naïve HBeAg positive chronic hepatitis B virus infection patients with normal ALT

**DOI:** 10.1080/21505594.2022.2158710

**Published:** 2023-01-04

**Authors:** Lu Zhang, Liu Yang, Yuanjiao Gao, Xiaoyue Bi, Yanjie Lin, Wen Deng, Tingting Jiang, Yao Lu, Hongxiao Hao, Gang Wan, Wei Yi, Yao Xie, Minghui Li

**Affiliations:** a Department of Hepatology Division 2, Beijing Ditan Hospital, Capital Medical University, Beijing, China; b Department of Hepatology Division 2, Peking University Ditan Teaching Hospital, Beijing, China; c Department of Medical Record Statistics, Beijing Ditan Hospital, Capital Medical University, Beijing, China; d Department of Gynecology and Obstetrics, Beijing Ditan Hospital, Capital Medical University, Beijing, China

**Keywords:** Aspartate aminotransferase, liver inflammation, nomogram, PLT, Hepatitis B e antigen

## Abstract

The purpose of this study was to develop an effective and non-invasive nomogram for evaluating liver obvious inflammation in untreated HBeAg positive patients with chronic hepatitis B virus (HBV) infection. A nomogram was established on a model cohort of 292 treatment-naïve HBeAg positive patients with normal alanine aminotransferase (ALT ≤40 U/L) at Beijing Ditan Hospital from January 2008 to March 2018. Then the nomogram was prospectively validated in a cohort of 88 patients from July 2019 to May 2021. Calibration curves and Concordance index were used to evaluate the accuracy of prediction and identification performance of the model. In untreated HBeAg positive chronic hepatitis B virus infection patients with normal ALT, the formula for predicting liver inflammation was Logit (P) =-0.91-0.41×log_10_ (qHBeAg)+0.11×AST-0.01×PLT. The nomogram had C-index of 0.751 (95% CI, 0.688–0.815), indicating a good consistency between prediction and real observation on the model cohort. The validation cohort confirmed its good performance. In this study, liver inflammation nomograms based on HBeAg, AST, and PLT were established and verified in treatment-naïve HBeAg positive chronic HBV patients with normal ALT.

## Introduction

About 240 million people were infected by the hepatitis B virus (HBV) over the world. End-stage liver disease and hepatocellular carcinoma are mainly related with chronic HBV infection in China. In chronic hepatitis B (CHB) patients, interferon and/or the oral antiviral drug- nucleo(s)tide analogues (NAs), have enabled more patients to get virological responses, and some preponderant patients have achieved clinical cure. As an indicator of liver inflammation, alanine aminotransferase (ALT) ≥ 2 ULN was used as the condition to start antiviral therapy in most guidelines [1–4]. Guidelines of WHO in 2015 and China in 2019 proposed that antiviral treatment could be considered when ALT >1 ULN (40 U/l) [5,6]. Some CHB patients with normal alanine aminotransferase (ALT) and age of less than 30 years old are in the immune tolerant-stage period and not recommended to treat [7]. With the update of the guidelines, it was recognized that if patients with normal ALT were not treated and only followed up for every 3 to 6 months, some with liver inflammation or fibrosis would be ignored. Liver pathology is regarded as a reliable standard for histological inflammation. However, liver biopsy is invasive and difficult to repeat, which limits its use. Therefore, it is necessary to explore a non-invasive prediction model of liver inflammation.

With the help of liver biopsy, this study aimed to establish a non-invasive liver inflammation prediction model in HBeAg positive chronic hepatitis B virus infection patients with normal ALT level by using gender, age, serological HBV markers, HBV DNA, ALT, AST, blood routine and *etc*.

## Patients and methods

### Enrollment criteria

We retrospectively included treatment-naïve HBeAg positive patients with chronic HBV infection in Beijing Ditan Hospital from January 1, 2008 to March 31, 2018. A nomogram was established to predict liver inflammation in the model cohort of 292 patients. A cohort of 88 patients was used for prospectively external validation from July 2019 to May 2021. The study was approved by the Ethical Department of Beijing Ditan Hospital, Capital Medical University (No.201703601) and registered on the Clinical Trials.gov (NCT04032275).

### Inclusion and exclusion criteria

HBeAg positive patients with chronic HBV infection for over 6 months, HBV DNA more than 10^3^ IU/ml and NALT ≤40 U/L (1 ULN) were enrolled. All patients had liver pathology speciments and had never received any antiviral therapy. Exclusion criteria: Patients with autoimmune hepatitis, alcoholic liver disease, drug-induced liver injury, other viral hepatitis (such as type A, C, D, E), or metabolic liver disease. Patients with HIV infection, liver cirrhosis, liver cancer, or pregnant people were also excluded. Liver cirrhosis was diagnosed according to Chinese guidelines by the liver pathology or clinical symptoms of liver disease, laboratory tests, liver imaging (B, CT, or MRI) and gastroscopy results [8]. The following data were collected: gender, age, qHBsAg, qHBeAg, HBV DNA level, liver function, and blood routine. Patients were divided into non-obvious inflammation group (<4 points) and obvious inflammation group (≥4 points) according to their pathological Knodell score of liver specimens. The detection interval of qHBsAg is 0.05–250 IU/ml. If the detected value of qHBsAg concentration is > 250 IU/ml, it will be diluted to 1:500.

### Statistical method

SAS 9.2 and R 3.02 software were adopted for statistical analysis. The data were described by median (Q1, Q3), means ± SD or frequency and rate. Student’s *t*-test for normal distribution data, Wilcoxon rank sum test for non-normal distribution data, or Fisher’s exact or χ^2^ for qualitative indicators were used for analysing intergroup differences. Logistic regression was adopted for analysing the factors related with inflammation. Both sides are tested, and *p* < 0.05 was considered significantly different.

### Establishment of prediction model

Age, gender, qHBsAg, qHBeAg, HBV DNA, ALB, TBIL, AST, ALT, PTA, WBC, and PLT were taken into the model cohort as independent variables, and significant liver inflammation (Knodell score ≥4) in pathology of liver biopsy was taken as dependent variable. Prediction formula was established using the model cohort.

### Liver inflammation nomogram

R 3.02 software was used for establishing nomogram to predict significant liver inflammation. The independent factors obtained from multivariate analysis were introduced into the model. Each variable was assigned to a specific value in the score axis. Total scores from adding each value corresponded to prediction probabilities on risk axis. As for the validation of predictive nomogram, we used calibration curve and C-index to evaluate the discriminant ability and prediction accuracy of nomogram. 1.0 of C-index represents complete consistency, indicating that the predicted probability of the model is completely close to the actual result, while 0.5 of C-index represents random chance. Bootstraps were used to eliminate overfitting.

## Results

### Baseline data of model cohort and validation cohort

In chronic hepatitis B virus infection patients, 292 were used to establish the model (model cohort) and 88 were used to prospectively validate the model (validation cohort). No differences were found in gender, Log_10_qHBsAg, Log_10_HBV DNA, ALB, TBil, AST, ALT, PLT, and WBC between the two cohorts (Table 1). There were significant differences in age (34.26 ± 9.77 years vs. 38.47 ± 9.19 years, *P* < 0.001), Log_10_qHBeAg (2.56 ± 0.97S/CO vs. 2.12 ± 1.19S/CO, *P* = 0.002), and PTA (87.36 ± 8.90%, vs. 96.02 ± 10.61%, *P* <0.001) between model cohort and validation cohort. Age accounted for 63.70% (≥30 years) or 30.14% (≥40 years) in model cohort, 76.14% (≥30 years) or 44.32% (≥40 years) in validated cohort. The baseline data of non-significant liver inflammation group and significant liver inflammation group in the model cohort are summarized in Table 2. The age, Log_10_qHBeAg, Log_10_HBV DNA, AST, and PLT had obvious effects on significant liver inflammation (*P* < 0.001).

**Table 1.** Baseline characteristics of model cohort and validation cohort.

	All	Model cohort	Validation cohort	Statistics	*P* value
	(n = 380)	(n = 292)	(n = 88)		
Sex					
Male,n(%)	210(55.26)	170(58.22)	40(45.45)	4.46(*χ*^2^)	0.035
Female,n(%)	170(44.74)	122(41.78)	48(54.55)		
Age (years)	35.24 ± 9.79	34.26 ± 9.77	38.47 ± 9.19	−3.58(*t*)	<0.001
Log_10_qHBsAg (IU/ml)	3.43 ± 0.97	3.42 ± 0.96	3.46 ± 1.01	−0.40(*t*)	0.691
Log_10_qHBeAg (S/CO)	2.46 ± 1.04	2.56 ± 0.97	2.12 ± 1.19	3.14(*t*)	0.002
Log_10_qHBV DNA (IU/ml)	6.65 ± 1.66	6.73 ± 1.55	6.39 ± 1.96	1.46(*t*)	0.146
ALT (U/L)	28.0(21.5,33.5)	28.3(21.6,33.5)	27.1(20.9,33.3)	0.63(Z)	0.526
AST (U/L)	22.9(19.4,26.9)	22.7(19.3,26.3)	24.0(20.0,28.0)	1.62(Z)	0.105
TBIL (umol/l)	12.5(9.5,16.2)	12.7(9.5,16.1)	12.0(9.6,17.0)	0.71(Z)	0.480
ALB (g/L)	45.7(43.2,48.0)	45.7(43.5,48.2)	45.8(43.0,47.0)	1.42(Z)	0.156
WBC (10^9^/L)	5.8(4.8,6.7)	5.8(5.0,6.8)	5.7(4.2,6.5)	1.90(Z)	0.058
PLT(10^9^/L)	205.58 ± 55.12	203.23 ± 52.78	213.37 ± 61.94	−1.51(*t*)	0.131
PTA (%)	89.71 ± 10.14	87.36 ± 8.90	96.02 ± 10.61	−6.74(*t*)	<0.001

Note: Data are quartile division or mean±SD for continuous factors. The n (%) was used for categorical factors.

**Abbreviations**: qHBsAg, quantitative HBsAg; qHBeAg,quantitative HBeAg; ALT, alanine aminotransferase; AST, aspartate aminotransferase; PTA, prothrombin activity. WBC, white blood cell; TBil, total bilirubin; ALB, albumin; PLT, platelet.

**Table 2.** Comparison between non-significant liver inflammation group and significant liver inflammation group in the model cohort.

	Model cohort	non significant liver inflammation group	Significant liver inflammation group	Statistics	*P* value
	(n = 292)	(n = 204)	(n = 88)		
Sex					
Male,n(%)	170(58.22)	127(62.25)	43(48.86)	4.53(*χ*^2^)	0.033
Female,n(%)	122(41.78)	77(37.75)	45(51.14)		
Age (years)	34.26 ± 9.77	32.69 ± 8.81	37.92 ± 10.92	−3.97(*t*)	0.000
Log_10_qHBsAg (IU/ml)	3.42 ± 0.96	3.43 ± 1.01	3.38 ± 0.81	0.46(*t*)	0.648
Log_10_qHBeAg (S/CO)	2.56 ± 0.97	2.76 ± 0.85	2.08 ± 1.05	5.38(*t*)	0.000
Log_10_qHBV DNA (IU/ml)	6.73 ± 1.55	6.96 ± 1.50	6.19 ± 1.55	3.92(*t*)	0.000
ALT(U/L)	28.3(21.6,33.5)	27.5(21.4,33.1)	29.2(23.7,34.8)	1.85(Z)	0.064
AST(U/L)	22.7(19.3,26.3)	21.5(18.4,24.5)	25.3(21.7,30.3)	5.66(Z)	<0.001
TBIL(umol/l)	12.7(9.5,16.1)	12.9(9.6,16.5)	12.6(9.5,15.2)	1.03(Z)	0.304
ALB (g/L)	45.7(43.5,48.2)	46.2(44.1,48.4)	45.0(42.2,47.3)	2.79(Z)	0.005
WBC (10^9^/L)	5.8(5.0,6.8)	5.8(5.0,6.8)	5.8(4.9,6.7)	0.38(Z)	0.707
PLT (10^9^/L)	203.23 ± 52.78	211.96 ± 52.47	183.01 ± 47.99	4.44(*t*)	0.000
PTA (%)	87.36 ± 8.90	87.30 ± 9.25	87.48 ± 8.12	−0.14(*t*)	0.885

Note: Data are quartile division or mean±SD for continuous factors. The n (%) was used for categorical factors. **Abbreviations**: qHBsAg, quantitative HBsAg; qHBeAg, quantitative HBeAg; ALT, alanine aminotransferase; AST, aspartate aminotransferase; PTA, prothrombin activity. WBC, white blood cell; TBil, total bilirubin; ALB, albumin; PLT, platelet.

### Single and multiple logistic regression analysis

The baseline data from the model cohort was used for single and multiple factors logistic regression analysis. Gender, age, Log_10_qHBsAg, Log_10_qHBeAg, Log_10_HBV DNA, ALB, TBil, AST, ALT, PTA, WBC, and PLT were introduced into single-factor analysis. The results showed that age, Log_10_qHBeAg, Log_10_HBV DNA, AST, and PLT had significant effects on significant liver inflammation (*P* <0.001, Table 3). These five factors were then introduced into multiple factors logistic regression analysis. Log_10_qHBeAg, PLT and AST were found to be independent variables of significant liver inflammation (Table 4). Log_10_qHBeAg and PLT were protective factors (OR = 0.67 and OR = 0.99, respectively), and AST was risk factor (OR = 1.11). The formula for evaluating obvious liver inflammation is: Logit(P)=-0.91-0.41×Log_10_ (qHBeAg)+0.11×AST-0.01×PLT.

**Table 3.** Univariate logistic regression analysis of factors for obvious liver inflammation in the model cohort.

						95%CI for OR	
	b	stb	Wald	P	OR	Lower	Upper
Sex	0.55	0.26	4.49	0.034	1.73	1.04	2.86
Age (years)	0.06	0.01	16.63	<0.001	1.06	1.03	1.09
Log_10_qHBsAg (IU/ml)	−0.06	0.14	0.18	0.675	0.94	0.76	1.24
Log_10_qHBeAg (S/CO)	−0.69	0.13	27.44	<0.001	0.50	0.39	0.65
Log_10_HBV DNA (IU/ml)	−0.30	0.08	13.54	<0.001	0.74	0.63	0.87
ALT(U/L)	0.03	0.02	3.68	0.055	1.03	1.00	1.07
AST(U/L)	0.14	0.03	30.57	<0.001	1.15	1.10	1.21
TBIL(umol/l)	−0.03	0.02	2.28	0.131	0.97	0.93	1.01
ALB (g/L)	−0.04	0.02	3.16	0.076	0.96	0.91	1.00
WBC(10^9^/L)	−0.02	0.09	0.07	0.789	0.98	0.82	1.16
PLT(10^9^/L)	−0.01	0.00	17.60	<0.001	0.99	0.98	0.99
PTA (%)	0.00	0.02	0.02	0.884	1.00	0.97	1.03

**Abbreviations**: qHBsAg, quantitative HBsAg; qHBeAg, quantitative HBeAg; ALT, alanine aminotransferase; AST, aspartate aminotransferase; PTA, prothrombin activity. WBC, White blood cell; TBil, total bilirubin; ALB, albumin; PLT, platelet.

**Table 4.** Multivariate logistic analysis of significant liver inflammation in the model cohort.

						95%CI for OR	
	b	stb	Wald	P	OR	Lower	Upper
Intercep	−0.91	1.00	0.82	0.364			
Log_10_qHBeAg (S/CO)	−0.41	0.15	7.56	0.006	0.67	0.50	0.89
AST(U/L)	0.11	0.03	15.21	0.000	1.11	1.05	1.17
PLT(10^9^/L)	−0.01	0.00	5.71	0.017	0.99	0.99	1.00

**Abbreviations**: qHBeAg, quantitative HBeAg; AST, aspartate aminotransferase; PLT, platelet.

### Scoring system for significant liver inflammation

The scoring system of significant liver inflammation was established based on the multiple factors logistic regression analysis. A scoring system was developed according to the parameters and coefficients in the formula. The coefficient of each variable in the formula was transformed to corresponding score for the point assignment in scoring system. In the scoring system, each Log_10_qHBeAg, AST, and PLT value has a specific corresponding point, and points of the three were added to obtain the total score. The corresponding risk of the total score was the probability (0–1) of significant liver inflammation. For example, if the total point was above 16, the probability of significant liver inflammation was predicted to be 0.7, that is, 70% (Table 5).

**Table 5.** Point assignment and risk of the scoring system on significant liver inflammation.

AST (U/L)	Points	PLT (10^9^/L)	Points	Log_10_qHBeAg (S/CO)	Points	Total Points	Risk
5	0	100	7	0	4	6	0.05
10	1	150	6	0.5	3	8	0.1
15	3	200	5	1	3	10	0.2
20	4	250	4	1.5	2	12	0.4
25	6	300	3	2	2	13	0.5
30	7	350	2	2.5	1	14	0.5
35	9	400	1	3	1	16	0.7
40	10	450	0	3.5	0	18	0.8
						20	0.9

**Abbreviations**: qHBeAg, quantitative HBeAg; AST, aspartate aminotransferase; PLT, platelet.

### Establishment and verification of the significant liver inflammation nomogram

**Figure 1.**
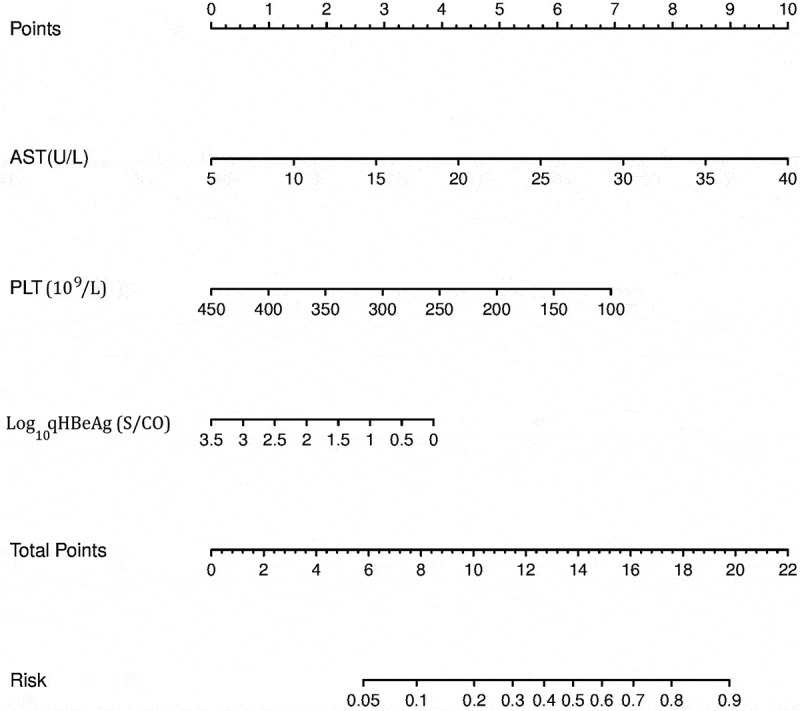
Nomogram for predicting significant liver inflammation based on model cohort. Abbreviations: qHBeAg, quantitative HBeAg; AST, aspartate aminotransferase; PLT, platelet.

The baseline values of AST, PLT, and Log_10_qHBeAg of all patients were used to predict significant liver inflammation. In the nomogram, AST, PLT, and Log_10_qHBeAg values were, respectively, located on variable axis, and a vertical line was drawn upward to determine a score on the points axis. The total score came from the sum of the score of AST, Log_10_qHBeAg and PLT (Figure 1). The total scores determine the prediction probability on the risk axis by drawing a downward vertical line. The higher the total score was, the higher the prediction probability of liver inflammation was. If detection values of a chronic HBV infection patient are 40 U/L for AST, 150 × 10^9^/L for PLT, 3.5 S/CO for Log_10_ qHBeAg, the corresponding score is 10, 6, and 0 in the points axis, respectively. The total score from AST, PLT, and Log_10_qHBeAg is 16, resulting in a corresponding risk of 0.7 in the risk axis, that is, the probability of liver inflammation is 70%.

C-index showed consistency between actual probability and predicted probability of the outcome. C-index revealed a good performance of 0.751, 95%CI (0.688–0.815) from the model cohort and 0.774, 95%CI (0.675–0.873) from validation cohort.

**Figure 2.**
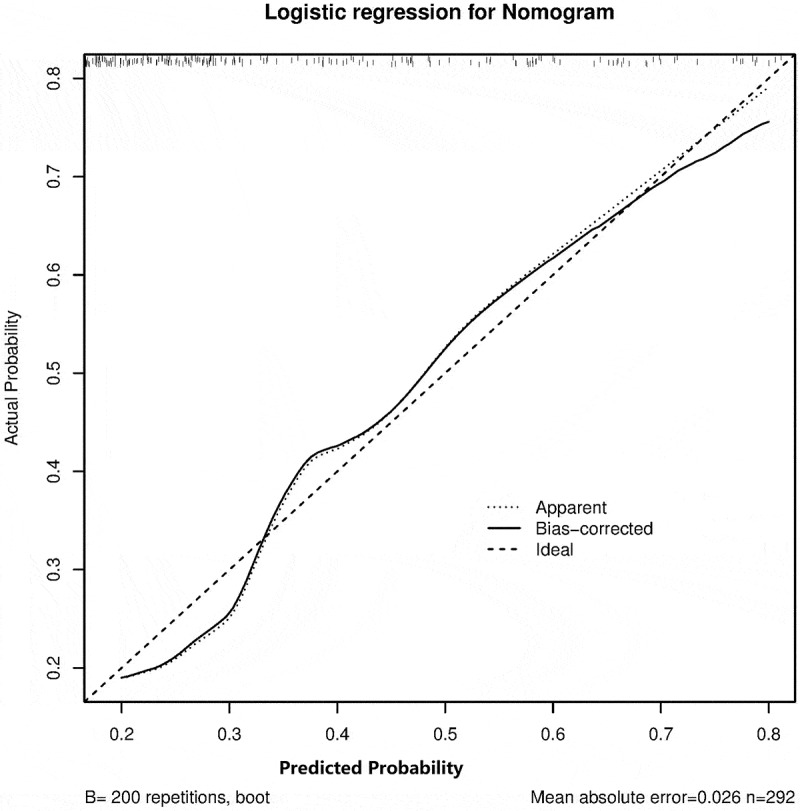
The calibration curve of significant liver inflammation nomogram on the model cohort. Predicted and actual liver inflammation probability were respectively plotted on the X-axis and the Y-axis. The 45-degree dashed lines through the coordinate origin represent the excellent calibration models. Bootstraps with 200 resamples were adopted.

**Figure 3.**
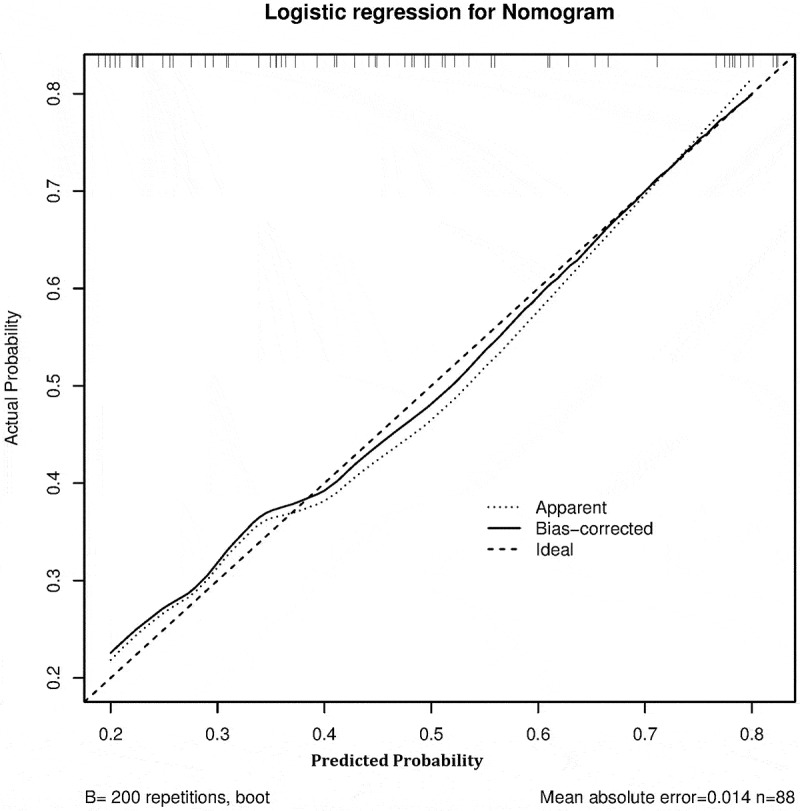
The calibration curve of significant liver inflammation nomogram on the validation cohort. Bootstraps with 200 resamples were adopted.

The method of calibration curve was adopted for the consistency of the model cohort and the validation cohort. The calibration curve showed that the prediction probability of the model was consistent with the actual probability, indicating that the prediction accuracy of the correction curve was good in both the model cohort (Figure 2) and validation cohort (Figure 3).

## Discussion

Study of the natural history of chronic HBV infection indicates that patients experience a process of gradual liver inflammation. Liver pathology is the most direct and accurate evaluation tool for liver inflammation. It remains unclear which non-invasive indicators are most likely to identify HBV natural history stages. In the present study, we established a non-invasive nomogram for predicting obvious liver inflammation in HBeAg positive chronic hepatitis B virus infection patients with NALT.

In treatment naive HBeAg positive chronic hepatitis B virus infection patients, normal ALT (≤40 U/L) does not mean no inflammation or fibrosis of liver. It has been found that HBV DNA is an intrinsic driving factor of CHB progression [9]. Even if ALT is normal and there is no liver fibrosis, there is obvious inflammation in 28.7% patients if serum HBV DNA can be detected [10]. Another study reported that 37% patients with NALT had obvious inflammation and/or fibrosis in liver [11]. Some NALT patients without anti-HBV treatment develop cirrhosis and liver cancer [12–14]. About 30%-73% patients died from cirrhosis or liver failure, and 53%-80% of HCC patients failed to meet the antiviral treatment indications [14]. On the other side, if patients in persistent immune tolerant stage are treated with antiviral therapy, it is difficult for them to obtain a complete virological response, and only 5% could achieve HBeAg seroconversion [15]. Therefore, patients who have normal ALT but need antiviral treatment should be distinguished from those in true immune tolerant stage. What’s more, it’s reported that 41% patients with relative high ALT level (26–40 U/L) had significant inflammation as compared to 20% of those with low ALT (<25 U/L) [11]. Thus, it’d be helpful to establish a non-invasive liver inflammation prediction model in patients with chronic HBV infection and normal ALT.

Some serum indicators may help determine the natural stages of chronic HBV infection instead of liver pathology. Most guidelines recommend that after evaluating HBeAg serum status, quantitative HBV DNA, and ALT level can help to distinguish immune clearance stage [1]. The quantification of qHBsAg and qHBeAg levels in natural state changes with the virus natural stages and host immune response [7,16–18]. *In vivo*, HBV DNA replication is originated from cccDNA in hepatocytes [19]. In treatment-naïve HBeAg positive patients, serum HBV DNA, qHBsAg, and qHBeAg are all associated with cccDNA and intrapathological HBV DNA [20]. qHBsAg and qHBeAg tend to gradually decrease with ageing, and liver inflammation is negatively correlated with qHBsAg and qHBeAg [21]. The levels of qHBsAg and qHBeAg could help distinguish immune clearance in HBeAg positive patients [16,21–23].

The characteristics of immune tolerant stage involve ALT, HBsAg, and HBV DNA [17]. Other studies also found that qHBsAg and qHBeAg in treatment-naïve HBeAg positive patients were correlated with serum HBV DNA [17,24–27]. Serum HBsAg level may reflect the number and transcriptional activity of cccDNA in hepatocytes [28]. HBsAg and HBeAg can be used to distinguish the immune tolerant period and monitor the efficacy of antiviral therapy and predict functional cure [21,29–31]. Besides qHBsAg, qHBeAg, HBV DNA, and ALT, we also included WBC, PLT and age as candidate indicators for establishing the prediction model. In the process of liver fibrosis or cirrhosis, there is gradual enlargement of spleen, and decrease in the white blood cell (WBC) and PLT [32]. With the increase of age, 10%–15% of CHB patients break the immune tolerant stage every year [1].

After univariate statistical analysis, liver inflammation indexes with statistically significance (*P* <0.01) were included in the multivariate analysis. The results showed that PLT, AST and HBeAg were independent influencing variables of liver inflammation according to the multivariate logistic analysis. The influence of age on liver inflammation was statistically significant by univariate analysis (*P* < 0.001), but failed to enter the prediction model. It is common for AST and PLT to be incorporated into liver disease models such as APRI and FIB-4, which are recognized as prediction models related to liver fibrosis [33]. In the population with normal ALT, although the PLT count is normal, it decreases gradually by 130,000/mm^3^ or less, which is also regarded as one of the signs of disease progression that suggests anti HBV treatment [14]. It seems that AST can better predict significant liver inflammation [34]. Many studies have found that qHBeAg and qHBsAg are inversely proportional to liver inflammation [22,23]. Our previous study found that HBeAg may be not inferior to HBsAg on predicting of liver inflammation [21]. Serum qHBsAg and HBV DNA are jointly involved in the prediction model of cirrhosis and liver cancer by REVEAL-HBV [35]. S antigen comes from infectious Dane particles or from non-infectious spherical and filamentous sub-viral particles [36]. It can be derived from cccDNA transcription and translation or from HBV DNA integrated into the host genome [37]. The level of HBV integration into host genome was very low in the stage of HBeAg positive immune tolerant period [27]. These characteristics of S antigen may partially reduce its specificity as a biomarker for virus replication.

Our model for evaluating obvious inflammation in chronic HBV infection covers the indicators of inflammation, virus replication or immune status, and liver disease progression. It’s intuitive, simple, and had good performance in treatment-naïve HBeAg positive chronic hepatitis B virus infection patients as confirmed by external prospective verification using calibration curve and C-index, which can evaluate the correctness of prediction and identification performance of the model. The calibration curve was used for evaluating the closeness between the estimated risk of the prediction model and the real observation [38].

At present, the non-invasive prediction model for beginning anti-HBV treatment was based on liver pathological of inflammation or fibrosis. There are several models that can predict the progression of liver disease after chronic HBV infection, but most of them focus on predicting liver fibrosis, and few model was used for predicting liver inflammation [39]. In most cases, inflammation often precedes fibrosis, and repeated liver inflammation eventually leads to fibrosis. The anti-HBV treatment prediction model TREAT-b was used for African with A and E genotypes, but Chinese populations are dominated by genotypes B and C [40]. In this study, the prediction liver inflammation formula was further simplified into a scoring system and nomogram. It can be used for long-term monitoring of liver inflammation, so as to help to determine treatment strategies.

This study has several limitations. First, it is a cross-sectional study. If the patients can be followed up for a long time, it will have greater significance. Second, there is no provision score matching (PSM) by gender and age. Third, some new and promising predictive factors may be introduced into the prediction model in the future, such as hepatitis B core-related antigen (qHBcrAg)/hepatitis B core antibody (HBcAb), whose reagent licences in China is limited to scientific research applications now.

In conclusion, for the untreated HBeAg positive patients with chronic HBV infection and normal NALT (≤40 U/L) in China, we established and verified a non-invasive nomogram model based on HBeAg, AST, and PLT to predict significant liver inflammation.

## Data Availability

The data used to support the findings of this study are available from the corresponding author upon request.

## Abbreviations

ALB, albumin; ALT, alanine aminotransferase; APRI, aspartate aminotransferase-to-platelet ratio index; AST, aspartate aminotransferase; CHB, chronic hepatitis B; cccDNA, covalently closed circular DNA; DILI, Drug-induced liver injury; FIB-4, Fibrosis 4 Score; HBcrAg, Hepatitis B virus core-related antigen; NALT, normal alanine transaminase; PLT, platelet; PTA, prothrombin activity; qHBeAg, quantitative HBeAg; qHBsAg, quantitative HBsAg; WBC, White blood cell; TBil, total bilirubin.
